# Strong Correlation between HLA and Clinical Course of Subacute Thyroiditis—A Report of the Three Siblings

**DOI:** 10.3390/genes11111282

**Published:** 2020-10-29

**Authors:** Magdalena Stasiak, Andrzej Lewiński

**Affiliations:** 1 Department of Endocrinology and Metabolic Diseases, Polish Mother’s Memorial Hospital-Research Institute, 93-338 Lodz, Poland; mstasiak33@gmail.com; 2 Department of Endocrinology and Metabolic Diseases, Medical University of Lodz, 93-338 Lodz, Poland

**Keywords:** subacute thyroiditis, human leukocyte antigen (HLA), recurrence, Graves’ disease

## Abstract

Subacute thyroiditis (SAT) is a thyroid inflammatory disease with susceptibility associated with the presence of human leukocyte antigen (HLA)-B*35, -B*18:01, -DRB1*01 and -C*04:01. Previous viral infection is considered as a triggering factor in genetically predisposed individuals. The influence of HLA on the SAT course was previously suggested. We aim to present the three siblings—female twins and their brother—with very close onset but different clinical courses of SAT, which appeared to be HLA-dependent. The HLA profile in the reported three siblings is strongly correlated with both SAT and Graves’ disease (GD), however the coexistence of particular sets of high risk and protective alleles seems to be crucial for the GD development and the SAT course. The co-occurrence of HLA-DRB1*15:01 and/or -B*07:02, possibly together with the lack of HLA-A*01:01 and -B*41:01 seems to be key factors protecting against the development of GD with high TRAb levels, as well as against the recurrent SAT course and steroid dependence.

## 1. Introduction

Subacute thyroiditis (SAT) (also called granulomatous thyroiditis, giant cell thyroiditis or de Quervain’s thyroiditis) is a thyroid inflammatory disease with susceptibility associated with the presence of human leukocyte antigen (HLA)-B*35, -B*18:01, -DRB1*01 and -C*04:01 [1]. Previous viral infection (occurring approximately 2–6 weeks earlier) is considered as a triggering factor in genetically predisposed individuals. The prevalence of SAT is the highest in middle aged women, and female patients account for 75–80% of individuals with the disease [2]. The most common presentation is the anterior neck pain radiating ipsilaterally up to the jaw and ear, and down to the upper part of the chest. However the painless course has been more and more often reported [2,3,4]. Fever is often present, reaching frequently over 39 °C, rising especially at night. Patients usually complain of asthenia and malaise, and some symptoms of thyrotoxicosis may be present. Laboratory findings include characteristically high erythrocyte sedimentation rate (ESR). C reactive protein (CRP) level is also elevated, however this parameter is less specific for SAT. Laboratory markers of hyperthyroidism are often present. The level of thyroid antibodies is normal in most patients. However, in recent years, the presence of thyroid peroxidase antibodies (aTPO) and/or thyroglobulin antibodies (aTg) was found in one third of the SAT patients, and the coexistence of thyrotropin receptor antibodies (TRAb) was demonstrated in 6% of SAT cases [2]. The ultrasound (US) features of SAT include hypoechoic and heterogeneous areas with blurred margins, poorly vascularized on color Doppler [5,6,7]. In Caucasian patients, the recurrence rate is approximately 14% [8].

The aim of the study is to present the three siblings—female twins and their brother—with very close onset but different clinical courses of SAT, which seemed to be HLA-dependent. In the general population, the influence of HLA on the SAT course was described [8], but has never been so clearly pronounced as it is in this family. We aimed to report the unique case of the three siblings in whom a direct significance of HLA background dominates over other factors, including environmental ones.

## 2. Case Presentation

### 2.1. Patients’ Description

As the first of the siblings, a 34-year-old male (MO) was referred to our department due to severe neck pain, fever and thyrotoxicosis. The diagnosis of SAT was made on the basis of the criteria proposed recently by our study team [9]. Clinical characteristics and the laboratory results of all the patients are presented in Table 1. Due to the severe clinical course, treatment with prednisone was introduce with the permanent relief of the symptoms.

The following year in August the same symptoms occurred in his 31-year-old sister (DO), who lived with her twin-sister (KO), separately from MO. Clinical characteristics and laboratory results were similar as in her brother (Table 1), and after prednisone therapy the permanent cure was achieved.

In the meantime, in June, the neck pain and fever occurred in KO (the twin sister of DO). KO was diagnosed with SAT in our department and the same treatment was introduced due to very severe neck pain and fever. However, her clinical course and laboratory results were different from MO and DO (Table 1). She was the only one who had preceding upper respiratory tract infection and was unnecessarily treated with azitromicin when the fever and neck pain occurred three weeks later. She had normal thyroid hormone levels while her TRAb level was significantly increased. Prednisone treatment (40 mg daily) was administered but every attempt to decrease the dose below 15 mg daily resulted in the recurrence of clinical symptoms and worsening of ultrasound (US) image and laboratory SAT markers. She had three episodes of recurrence and her SAT was diagnosed as glucocorticoid-dependent. Prednisone therapy was successfully withdrawn only after 11 months of constant treatment with different doses. Her thyroid hormone levels were normal throughout the treatment. After steroid withdrawal, the TRAb level increased up to 36.5 IU/L while thyroid function tests were as follows: thyrotropin (TSH) 2.48 uIU/mL, free thyroxine (FT4) 1.44 ng/dL (reference range 0.93–1.7), free triiodothyronine (FT3) 2.83 pg/mL (reference range 2.6–4.40). That time, her ESR was 26 (normal results < 15) but all her symptoms finally resolved. Her US pattern was typical for SAT but not for Graves’ disease.

Taking into account the close onset-time in all cases, different clinical courses in KO comparing to her twin-sister and her older brother, we performed high-resolution HLA typing to search for potential genetic reason of such SAT presentation in KO. DO was demonstrated to have identical HLA alleles as MO, but not as her twin-sister KO. The genetic SAT susceptibility alleles were the same in all the three—HLA-B*35:03 and -C*04:01. However, there were differences in the occurrence of other alleles, and the alleles associated with Graves’ disease in the Caucasian population were also present. The HLA results are presented in Table 2.

### 2.2. Material and Methods

The SAT diagnostic criteria were as follows: elevation of ESR (or at least CRP) plus hypoechoic area/areas with blurred margin and decreased vascularization in US plus FNAB confirmation of SAT, or at least FNAB exclusion of malignancy, plus at least one of the following: hard thyroid swelling and/or pain and tenderness of the thyroid gland/lobe and/or elevation of serum FT4 and suppression of TSH and/or decreased radioiodine uptake (RAIU) [9].

The presence of elevated TRAb concentration was the main criterion for the diagnosis of Grave’s disease (GD) [10,11].

DNA was extracted from the peripheral blood. *HLA-A*, *-B*, *-C*, *-DQB1* and *-DRB1* were genotyped using a next-generation sequencing method on Illumina platform (Illumina, San Diego, CA, USA). Sequencing-based HLA typing of the HLA genes *-A*, *-B*, *-C*, *-DQB1* and *-DRB1* was carried out in 96-well format within a semi-automated workflow by using MiaFora Flex5 typing kits (Immucor, Peachtree Konars, GA, USA). Long-range PCR amplification of five HLA loci was performed on DNA extracted from blood samples. Results of sequencing were analyzed by MiaFora NGS software. Data were considered sufficient whenever the coverage reached 40 and number of cReads exceeded 50,000. The sequencing included the most extensive coverage of the HLA genome, especially with respect to the five loci.

Serum levels of TSH and FT4 were measured by electrochemiluminescence immunoassay (ECLIA), Cobas e601 analyzer (Roche Diagnostics, Indianapolis, IN, USA), ESR was determined with Ves-Matic Cube 30 (Diesse, Monteriggioni, Tuscany, Italy), CRP was determined by VITROS^®^ 4600 Chemistry System (Ortho Clinical Diagnostics, Raritan, NJ, USA). Ultrasound examination was performed using a 7–14-MHz linear transducer (Toshiba Aplio XG; Toshiba, Japan). Fine needle aspiration biopsy (FNAB) was performed in all the three SAT patients using a 23-gauge needle. Smears were cytologically evaluated, and the presence of multinucleated giant cells together with mononucleated macrophages, and follicular epithelial cells against acute and chronic inflammatory dirty background (comprising of cellular debris and mixed inflammatory cells) was considered as a result typical for SAT.

### 2.3. Consent Procedures

All the three patients gave their informed written consent for all the procedures performed. The study was accepted by the Bioethical Committee of the Polish Mother’s Memorial Hospital—Research Institute, approval code 22/2016.

## 3. Discussion

The current paper presents the three siblings—female twins and their brother—with very close time of SAT onset, among whom in one of the female twins the clinical course was completely different with multiple episodes of recurrence and steroid dependence. In the second female twin and in their older brother, the SAT clinical course was typical, with neck pain, fever, moderate clinical and biochemical thyrotoxicosis and excellent response to steroid therapy. Interestingly, both of them reported no preceding viral infection, although DO lived together with KO. KO reported the upper respiratory infection with rhinorrhea, fever and cough approximately 3 weeks before SAT onset.

SAT is four times more common in females than males [2]. In our cases, the first one affected was the male, with the females—KO and DO—being affected 8 and 10 months later, respectively. A strong triggering factor must have activated the SAT development in all the three siblings during such a short period of time. The same viral infection could be considered although MO did not live with his sisters and DO and MO did not have infection symptoms. However they met several times and the time lag between SAT onset in MO and KO was much longer than between KO and DO who lived together. Thus, some viral factor should be taken into account as a triggering factor, with possible asymptomatic course in the two cases. KO was also the only one who was treated with antibiotic due to the presence of fever and neck pain just a few weeks after upper respiratory tract infection. Although antibiotic overuse resulting from misdiagnoses of SAT is an important problem, it does not influence the course of SAT because SAT is triggered by a viral infection [12].

Additional important difference was the presence of increased TRAb levels in KO, coexisting with normal thyroid hormone levels throughout the whole time of SAT duration. After three recurrence episodes, her TRAb level was five times higher than at the SAT onset. This was probably due to the fact that it was SAT that initiated the production of TRAb from the very beginning of the disease. This assumption can be supported by the thyroid US pattern, which in KO—identically as in her siblings—was typical for SAT, with no characteristic signs of Graves’ disease (GD). It is believed that SAT may trigger autoreactive B cells to produce TRAb, resulting—in some patients—in subsequent TRAb-associated thyroid dysfunction [4,13]. This hypothesis can be confirmed by the fact that there have been much more case reports of TRAb occurrence after SAT resolution than of the simultaneous presence of SAT and TRAb [14,15,16,17]. Perhaps this is mainly due to the lack of assessment and monitoring of TRAb levels during the course of SAT. It should also be indicated that the co-presence of TRAb in SAT patients was found not to influence the clinical course or the severity of thyrotoxicosis in SAT [2]. The clinical course of SAT in KO might be considered atypical because of the lack of thyrotoxicosis resulting from the destructive thyroiditis. In such a situation, we should consider the TRAb as neutral ones. However, we cannot exclude that the normal thyroid hormone levels resulted from overlapping of the destructive process in SAT and the presence of thyroid-blocking TRAb. Normal thyroid hormone levels after SAT resolution support the hypothesis of the presence of neutral TRAb in KO. However, TRAb can switch their character from blocking to a neutral or stimulating one. This is the crucial reason why the presence of hyperthyroidism is no longer considered as the main criterion of GD diagnosis [10,11]. Thus, the potential influence of TRAb switching should also be taken into account when analyzing thyroid hormone and TRAb levels in KO. Unfortunately, TRAb were not analyzed in our three siblings separately as thyroid stimulating immunoglobulins (TSIs) or thyroid blocking immunoglobulins (TBIs), but it should be suspected that there was a dominance of TBIs. The presence of TBIs in an active SAT case with normal, increased or decreased level of thyroid hormones has been described before [18,19]. Despite the increased TRAb levels, other thyroid antibodies (aTg, aTPO) were normal in KO. One could expect at least an increased aTg level as a simple consequence of the release of thyroid antigens due to the gland damage during SAT [2]. Presumably, genetic susceptibility is the most important factor determining the consequent development of autoimmunity after SAT.

Subacute thyroiditis is associated with the presence of particular HLA antigens, with HLA-B*18:01, -DRB1*01 and -C*04:01 having been recently reported in addition to the previously known HLA-B*35 [1]. Additionally, the simultaneous presence of HLA-B*18:01 and -B*35 was demonstrated to increase the risk of SAT recurrence [8]. The HLA-related differences were supposed to be the key factors explaining the differences in the clinical course of SAT [8]. Surprisingly, DO was demonstrated to have identical HLA profile as MO, but not with her twin-sister KO who had recurrent course of SAT and increased TRAb level. All of the three siblings were carriers of the same alleles associated with SAT susceptibility—HLA-B*35:03 and -C*04:01. These alleles could not be considered as independent risk factors as there is a linkage disequilibrium between them (i.e., these two alleles commonly occur together due to the close location of their loci). None of the siblings carried HLA-B*18:01, thus the influence of the alleles associated with high recurrence risk—i.e., co-presence of HLA-B*35 and -B*18:01—was excluded in KO. However, it is known that these alleles are not present in all patients with SAT recurrences. In some of them, other factors are critical for SAT relapse. Increased aTPO level and more severe thyrotoxicosis during the active phase of SAT was more often observed in patients without recurrences while slight thyrotoxicosis or normal thyroid hormone levels—like in KO—were more frequent in patients with recurrences [8]. This phenomenon can result from the presence of some other HLA alleles which potentially may play a protective role against SAT and/or its recurrence. Unfortunately, up to now, no allele was proven to be SAT-protective with satisfactory statistical significance [8].

However, among the three siblings there were significant differences in the occurrence of other alleles, which seems to play a more important role than was previously suspected. In all the three siblings, alleles HLA-DRB1*03:01 and -DQB1*02:01 associated with GD in the Caucasian population [20,21,22] were present. These two alleles cannot be considered as independent risk factors as there is a linkage disequilibrium between them [23]. KO was the only one demonstrated to carry also HLA-B*41:01 (-Bw6 in serological nomenclature) associated with increased risk of GD as well as HLA-A*01:01 which is supposed to be correlated not only with GD itself, but also with its persistent course [24,25]. Therefore, the HLA-related susceptibility to GD was more pronounced in KO than in MO or DO.

On the other hand, HLA-DRB1*15:01 and -B*07 were postulated to be protective against GD [26,27]. These alleles were present in MO and DO, but not in KO, who had high TRAb levels and recurrent SAT course. In the further follow up, MO and DO have never had increased TRAb levels, despite genetic high risk associated with the presence of HLA-DRB1*03:01 and -DQB1*02:01.

Therefore, the co-occurrence of HLA-DRB1*15:01 and/or -B*07:02, possibly together with the lack of HLA-A*01:01 and -B*41:01, seems to be key factors protecting MO and DO against the development of GD-related high TRAb levels, and—possibly—also recurrent course of SAT and steroid dependence.

The HLA profile in the reported three siblings is strongly correlated with both SAT and GD, however the coexistence of particular sets of high risk alleles and protective ones seems to be crucial for the GD development and the SAT course. The influence of HLA on some clinical features of SAT and GD was previously reported. As it was already indicated, particular HLA alleles are associated with high risk of SAT recurrence [8]. Moreover, sonographic pattern of SAT was demonstrated to be HLA-dependent, with HLA-B*18:01 being the key factor changing the SAT US image [5]. Some details of the clinical course of GD were also demonstrated to depend on HLA antigens. Vita et al. [28] reported that HLA haplotype influenced the onset age and the severity of hyperthyroidism in GD.

## 4. Conclusions

The different course of the disease in the patient with different HLA haplotype, despite the same potential viral triggering factor suspected on the basis of the SAT onset close to her siblings, indicates the crucial role of HLA in determining SAT clinical course. The occurrence of one particular allele is rarely decisive, and most frequently, the coexistence of several alleles determines the clinical course of one disease. We have clearly demonstrated such a situation in the three siblings, in whom the SAT course and HLA profile were very clearly associated. Awareness of the correlation between HLA and the SAT clinical course seems to be very important for disease management, and the treatment should be tailored based on the recurrence risk.

## Figures and Tables

**Table 1:** Clinical characteristics and laboratory results at the subacute thyroiditis (SAT) diagnosis.

Analyzed Parameter	MO	KO	DO
Gender	Male	Female	Female
SAT diagnosis date	October 2016	June 2017	August 2017
TSH (*n*: 0.27–4.2 mIU/L)	**0.01**	1.07	**0.01**
FT3 (*n*: 2.6–4.4 pg/mL)	**5.64**	3.27	**7.78**
FT4 (*n*: 0.93–1.7 ng/dL)	**2.43**	1.17	**3.02**
aTPO (*n*: < 34 IU/mL)	7.25	13.06	8.36
aTg (*n*: < 115 IU/mL)	10	114.5	73.02
TRAb (*n*: < 1.75 IU/mL)	0.56	**5.38**	0.48
ESR (*n*: < 12 mm/h)	**93**	44	**84**
CRP (*n*: < 1 mg/dL)	**8.11**	1.1	**3.91**
Neck pain	yes	yes	yes
Fever	yes	yes	yes
Sonographic pattern	Several hypoechoic areas	Several hypoechoic areas	Several hypoechoic areas
Preceding infection	no	yes	no
Antibiotic treatment before SAT diagnosis	no	yes (azitromicin)	no
Recurrence	no	Yes—3 episodes	no
Living together	no	yes	yes

The laboratory results out of the normal range are presented in bold. Abbreviations: aTg, thyroglobulin antibodies; aTPO, thyroid peroxidase antibodies; CRP, C-reactive protein; ESR, erythrocyte sedimentation rate; FT3, free triiodothyronine; FT4, free thyroxine; *n*, normal values; TRAb, thyrotropin receptor antibodies, TSH, thyrotropin. MO: a 34-year-old male, DO: MO’s 31-year-old sister, KO: DO’s twin-sister.

**Table 2:** Human leukocyte antigen (HLA) genotyping results in the three siblings.

Initials	Gender	HLA-A	HLA-A	HLA-B	HLA-B	HLA-C	HLA-C	HLA-DRB1	HLA-DRB1	HLA-DQB1	HLA-DQB1
DO	F		03:01	07:02 ^3^	35:03:00 ^1^	04:01 ^1^	07:02	03:01:00 ^2^	15:01:00 ^3^	02:01:00 ^2^	05:01
MO	M		03:01	07:02 ^3^	35:03:00 ^1^	04:01 ^1^	07:02	03:01:00 ^2^	15:01:00 ^3^	02:01:00 ^2^	05:01
KO	F	01:01	03:01	41:01:00	35:03:00 ^1^	04:01 ^1^	07:01	03:01:00 ^2^		02:01:00 ^2^	

^1^ Alleles associated with SAT; ^2^ Alleles associated with Graves’ disease; ^3^ Alleles protective against Graves’ disease.

## Abbreviations

aTg	thyroglobulin antibodies
aTPO	thyroid peroxidase antibodies
CRP	C reactive protein
ECLIA	electrochemiluminescence immunoassay
ESR	erythrocyte sedimentation rate
FNAB	fine needle aspiration biopsy
FT3	free triiodothyronine
FT4	free thyroxine
GD	Graves’ disease
HLA	human leukocyte antigens
SAT	subacute thyroiditis
TRAb	thyrotropin receptor antibodies
TSH	thyrotropin
US	ultrasound
